# Reciprocal Relationship Between Parenting Styles and Interpersonal Personality in Chinese Adolescents

**DOI:** 10.3389/fpsyg.2021.740026

**Published:** 2021-10-07

**Authors:** Weiqiao Fan, Mengting Li, Xinyin Chen

**Affiliations:** ^1^Research Institute for International and Comparative Education, Shanghai Normal University, Shanghai, China; ^2^Faculty of Education, The University of Hong Kong, Pokfulam, Hong Kong, SAR China; ^3^Graduate School of Education, University of Pennsylvania, Philadelphia, PA, United States

**Keywords:** interpersonal personality, parenting styles, filial piety, reciprocal relationship, Chinese adolescents

## Abstract

This study examined the reciprocal relations between the parenting styles and adolescents' interpersonal personality in China. A total of 722 sixth-grade Chinese junior high school students reported their interpersonal relatedness (IR) personality trait and perceived parenting styles of their parents. Of these students, 411 completed the survey again in eighth grade. One parent of each student rated their parenting styles. The results indicated that perceived paternal rejection negatively predicted adolescents' IR 2 years later, whereas perceived paternal behavioral controlling positively predicted adolescents' IR 2 years later. IR also positively predicted perceived paternal warmth 2 years later. Significant reciprocal association between adolescents' IR and perceived maternal rejection was found. Parent-rated behavioral control negatively predicted adolescents' IR, whereas Parent-rated filial piety positively predicted adolescents' IR. The results were discussed in the Chinese context.

## Introduction

Personality is one of the most important individual dispositions. Numerous theories have been conducted to examine how personality develops and what influences it (Caspi et al., 2005; Specht, 2017). There is a growing interest in adolescent personality development (e.g., Schofield et al., 2012) due to the findings about its links with subjective well-being, school performance, and career development (e.g., Rogers et al., 2008; Poropat, 2009; Cheung et al., 2012; Li, 2021). Parent is one of the most important socializing agents interacting with child (Damon et al., 2006). Parenting style, which is defined as a climate in which the family functions and child-rearing or socialization occurs (Darling and Steinberg, 1993), is an important factor that may be associated with children's personality development (Shiner and Caspi, 2003).

The mechanisms for the relations between personality and parenting are often discussed from two major perspectives. First, according to the internal working model (Bowlby, 1980; Bretherton, 1990), children internalize the experience from the interaction with their parents and develop the “self” and carry it to other contexts, which eventually leads to a relatively stable personality. Consistent with this perspective, studies have indicated that parenting styles play a significant role in the development of individual personality (Coplan et al., 2009; Kitamura et al., 2009). Second, according to the environmental elicitation model (Belsky, 1984; Shiner and Caspi, 2003), children's characteristics may elicit specific parenting behaviors and shape parenting styles. There is emerging evidence indicating children's personalities may predict parenting styles (Prinzie et al., 2012; Hong et al., 2015; Skinner et al., 2019). In fact, a bi-directional relation between parenting and child characteristics has been highlighted in socialization literature as well (e.g., Bell, 1968; Dodge, 1990).

Theoretically, the internal working model of behavior and the environmental elicitation model are not mutually exclusive (Anaya and Pérez-Edgar, 2019). For example, the transactional model of development emphasizes the bidirectional unfolding of parents' and children's behavior and posits that children's development occurs through the continuous dynamic interplay between child's characteristics and parent's response (Sameroff, 1987; Sanson et al., 2018). This theory has been supported by some results concerning the relations between parenting and child functioning (e.g., personality development) (Van den Akker et al., 2014; Van Heel et al., 2019). For example, Van den Akker et al. (2014) showed that maternal warmth and children's benevolent and openness traits contributed to each other in a bi-directional manner. Nevertheless, further research is needed to explore reciprocal associations between different parenting styles and children's personality in order to better understand the mechanisms of socialization and human development as suggested by the internal working model and the environmental elicitation model (Anaya and Pérez-Edgar, 2019).

However, the existing studies of relations between parenting styles and personality have been conducted mainly in Western societies based on the Western models of intrapersonal personality (e.g., the five factor model, McCrae and Costa, 2008) and parenting styles (e.g., the three-dimensional framework of responsiveness, demandingness, and autonomy support; Baumrind, 1971; Prinzie et al., 2009; Huver et al., 2010; Kim et al., 2013; Fu and Markus, 2014; Zhong et al., 2020). As a result, little is known about the relations in non-Western societies. Developmental theorists (e.g., LeVine, 1974; Chao, 1994; Chen, 2000) have long argued that social and cultural contexts are likely to affect parenting, individual characteristics, and their relations. Therefore, it is necessary to examine the links between parenting styles and personality in different societies, which may provide valuable information about the socialization processes beyond the Western frameworks.

For example, an important indigenous notion in socialization in the Confucian doctrine, which is the primary ideological system in Chinese and some other East Asian societies, is filial piety (Chan et al., 2009). The principle of filial piety stipulates that child should fulfill parents' expectations, maintain absolute obedience to parents, and care for elders in the family whereas parents are responsible for teaching and disciplining their children (Hsu, 1981; Chen, 2014; Jorgensen et al., 2017). A major socialization task for parents is to encourage and help children to learn and display filial piety from childhood (Ho, 1996). Relatedly, interpersonal relatedness is highly valued in group-oriented Chinese and Asian societies (Chen, 2000). Accordingly, researchers have identified Interpersonal Relatedness (IR) as a typical personality factor that describes the orientation toward harmonious social interactions and avoidance of conflict (Cheung et al., 2001; Fan et al., 2011). Empirical studies have supported the validity of IR in explaining and predicting adolescents' developmental outcomes, such as loneliness, life satisfaction, and career development (e.g., Cheung et al., 2012; Wan and Cheung, 2016; Xie et al., 2016; Li et al., 2019; Li, 2021). In this 2-year longitudinal study, we attempted to investigate reciprocal associations between parenting styles, as rated by parents and adolescents, and adolescents' interpersonal personality in China.

### Parenting Styles and Adolescents' Personality

The contributions of parenting styles to the development of adolescents' personalities have been well-documented in the literature (e.g., Kitamura et al., 2009; Schofield et al., 2012). For example, in an American adolescent sample, Schofield et al. (2012) found that maternal and paternal warmth significantly predicted adolescent alpha personality traits (including high agreeableness and consciousness and low neuroticism) 2 years later. Researchers also found that parental acceptance was a significant predictor of self-rated openness in Chinese adolescents (Fan and Wu, 2009) and teacher-rated creative personality in South Korean children (Lim and Smith, 2008). Similarly, Kitamura et al. (2009) found that parental caring styles predicted children's novelty-seeking trait. Weiss and Schwarz (1996) found significant links between parenting styles and the Big Five traits: unengaged and authoritarian parenting styles predicted low scores on agreeableness and openness to experience or high scores on neuroticism in the US.

Regarding the effects of adolescents' personality on their parent's parenting styles, de Haan et al. (2012) and Egberts et al. (2015) found longitudinal evidence with Belgian adolescents that extraverted, benevolent (agreeable), and imaginative (open to experience) traits evoked high parental warmth but low parental overreactivity/psychological control 2 years later. In an African American sample, Skinner et al. (2019) found in a 3-year longitudinal study that the self-rated expressivity of youths positively predicted maternal and paternal warmth and conflict.

In addition, it has been argued that parenting styles of mothers and fathers may be associated with children's behaviors and characteristics in different manners (Chen et al., 2000; Winsler et al., 2005; Latzman et al., 2009). Milevsky et al. (2007) found that authoritative mothering was positively associated with self-esteem and life satisfaction and negatively associated with depression; however, authoritative fathering was only negatively associated with depression. In a meta-analytic review that based on 48 studies, Kawabata et al. (2011) reported that uninvolved parenting of mothers, but not fathers, was positively associated with relational aggression, whereas psychologically controlling parenting of fathers, but not mothers, was positively related to relational aggression.

### Parenting Style and Personality in Chinese Adolescents: The Present Study

Social context is believed to play an important role in shaping parenting and child-rearing practices (Chen-Bouck et al., 2019), which serve to transmit the values and ideals of a culture to the next generation (Super and Harkness, 1997). Thus, the relations between parenting styles and personality of adolescents can be fully understood only in the context of the society in which they are embedded (Szapocznik and Kurtines, 1993).

Researchers have found that the Western framework may not be sufficient in capturing Chinese parenting styles (Chao, 1994; Chen et al., 2000). For example, although Chinese parents tend to be more power-assertive and controlling than Western parents, parental power assertion and control are often associated with care, concern, and involvement in Chinese culture, which may be reflected in the notion of *Guan* (strict control based on care and concern) (Chao, 1994; Chen et al., 2000). In addition, as mentioned earlier, filial piety parenting represents an indigenous parenting style in Chinese families, which is different from overprotection in the Western literature (Szapocznik and Kurtines, 1993; Young et al., 2003).

With regard to personality, it has been argued that the Western models, such as the Big Five (McCrae and Costa, 2008), may not provide an adequate and relevant understanding of personality in other contexts (Kim et al., 2006). Cheung et al. (2011) recommended a combined emic-etic approach to “bridge the divide between mainstream and indigenous psychology and provide a comprehensive framework to understand universal and culturally variable personality dimensions” (p. 5). The Cross-Cultural (Chinese) Personality Assessment Inventory (CPAI; Cheung et al., 2001; Fan et al., 2011) has been used to assess the personalities of adult and adolescent populations with a combined emic-etic approach. In addition to the Western-derived Big Five factors, a personality trait known as IR was validated in a series of studies in Chinese samples. For example, it was found that IR significantly predicted life satisfaction among Chinese adolescents (Xie et al., 2016). IR and independent personality related to the Big Five model also significantly predicted Chinese adolescents' loneliness at the individual and group levels (Li et al., 2019).

Therefore, we attempted in this study to expand the research on the relations between parenting and personality in Chinese adolescents by including some culturally relevant aspects such as parental encouragement of filial piety and adolescents' IR. We posited that adolescents' IR is reciprocally associated with Western-based parenting styles (e.g., warmth and rejection as the types of responsiveness, control as the type of demandingness, and encouragement of independence as the type of autonomy support) and the indigenous Chinese parenting style of encouragement of filial piety. Adolescence is a critical period for personality development. Adolescents during this period actively engage in dynamic social processes in constructing their self-identity and understanding their relationships with others (Caspi et al., 2005). As important socialization agents, parents may exert significant influence on adolescents' personality and, at the same time, adolescents' characteristics and experiences may shape parenting styles (Belsky, 1984; Huver et al., 2010).

Many of the studies of parenting and personality have used one informant (child reports or parental reports) in assessments. Children's and parents' perceptions of parenting may reflect different perspectives (Hou et al., 2020). For example, Yan et al. (2021) found that parents reported higher levels of warmth and monitoring than the adolescents did. Therefore, we collected data on parenting from both parents and adolescents in the present study.

## Method

### Participants

A total of 722 sixth-grade students were recruited from four regular public junior high schools (40.8% female; *M*_age_ = 11.49, *SD*_age_ = 0.61) in Shanghai, China. The schools were comparable in their structure, curriculum, and conditions. At the first wave of data collection, 499 (43.69% mothers) parents also participated. From the original sample, 411 eighth-grade students (56.93% of the students from the first wave of data collection) and 276 parents (60.51% mothers) participated in the second wave of data collection 2 years later. Because the parenting measure was completed by different parents at different times (e.g., the mother in Grade 6 and the father in Grade 8) for 85 students, data on parent-rated styles for these adolescents were not included in the data analysis.

Forty-two students did not report paternal educational level and forty-four students did not report maternal educational level. Of the fathers, 48.8% had an education of middle school or lower, 30.6% had an education of high school or technical training school, and 20.6% had a college/university education or higher. Of the mothers, 56.6% had an education of middle school or lower, 27.6% had an education of high school or technical training school, and 15.8% had a college/university education or higher. Fifty-seven students did not provide information about fathers' income and eighty-four students did not report mother's income. The available data indicated that for fathers' income (per month), 15.9% had 3000 RMB or less, 36.5% had 3,000–5,000 RMB, 28.0% had 5,000–10,000 RMB, and 19.6% had over 10,000 RMB. For mothers' income (per month), 37.9% had 3,000 RMB or less, 38.4% had 3,000–5,000 RMB, 16.5% had 5,000–10,000 RMB and 7.2% had over 10,000 RMB.

### Measures

#### IR

Adolescents reported on their IR by filling out the CPAI for Adolescents (CPAI-A, Form B) (Cheung et al., 2008), which was developed for assessing adolescent personality in Chinese populations. The CPAI-A (Form B) is composed of 25 general personality scales. In the present study, only the eight personality scales (i.e., harmony, family orientation, relationship orientation, graciousness vs. meanness, interpersonal tolerance, self vs. social orientation, veraciousness vs. slickness, and social sensitivity) of the IR factor were used. These scales were assessed using 90 items that were answered in a yes-or-no format. The average score for the eight scales was calculated for IR. The items for IR reflected the relevance of interpersonal dimensions [e.g., “It is hard for me to get along with others” (harmony; reversely scored), “I find it hard to sense other people's true reactions” (social sensitivity; reversely scored)] in the conceptualization of personality across Western and Eastern societies (Lin and Church, 2004; Fan et al., 2011). Previous studies have reported good internal consistency reliability, test-retest reliability, and construct validity for the CPAI-A (Cheung et al., 2008; Li et al., 2019). In this study, the Cronbach's alpha of the IR factor was 0.86 in Grade 6 and 0.83 in Grade 8.

#### Parenting Styles

Adolescents reported their perceptions of paternal and maternal parenting styles separately by using a measure that Chen et al. (1998) adapted from the Children's Report of Parent Behavior Inventory (CRPR; Schaefer, 1965). The Chinese version of the CRPR has been used in research on parenting in China (e.g., Xu et al., 2005). The revised measure includes parenting dimensions of (1) warmth (five items; e.g., “speak to me in a gentle and kind manner”), which refers to displaying positive emotions, acceptance, and support in parent-child interactions; (2) independence (six items; e.g., “encourage me to be independent and not to rely on her”), which refers to providing support for children to be autonomous and independent in attitudes and behaviors; (3) rejection (one item; i.e., “when my mother/father is angry, she/he scolds me”), which refers to parental indifferent and rejecting attitudes toward children; (4) behavioral control (five items; e.g., “believes in having a lot of rules and sticking with them”), refers to parental regulation of children's behaviors through monitoring, supervision, and emphasis on child obedience, and (5) filial piety (six items; e.g., “told me to be filial to my parents and elders”), refers to cultivating children to fulfill parents' expectations, obey their wishes, care for parents and elders in the family, and maintain interpersonal harmony in the family. The subscale of filial piety was added in this study. Adolescents were asked to rate each items on a 5-point scale, ranging from 1 (*not at all true*) to 5 (*very true*) for their mother and father.

One parent of each student reported on parenting styles using a measure adapted by Chen et al. (1998) from the Block Child-Rearing Practices Report (Block, 1965), with a filial piety subscale added in this study. The revised measure assessed the parenting styles corresponding to those in the adolescent measure, including parental warmth (four items; e.g., “I speak to my children in a gentle and kind manner”), independence (six items; e.g., “In many things, I let my children make their own decisions”), rejection (four items; e.g., “When I was angry, I scolded my children”), behavioral control (four items; e.g., “I always ask about my children's activities outside of school”), and filial piety (eight items; e.g., “I told my children to be filial to parents and elders”). The 5-point Likert scale was used for reporting (1 = *not at all true*, 5 = *very true*). The reliabilities of the subscales are presented in Table 1, ranging from 0.60 to 0.83 at Grade 6, and from 0.70 to 0.89 at Grade 8.

**Table 1:** Descriptive statistics and reliabilities (Cronbach's α).

	**Grade 6**			**Grade 8**		
	***M***	***SD***	**α**	***M***	***SD***	**α**
IR	8.10	1.73	0.86	8.23	1.65	0.83
Warmth_M	3.77	.98	0.83	3.59	0.94	0.89
Independence_M	3.81	0.85	0.76	3.67	0.85	0.86
Rejection_M#	2.83	1.47	–	2.78	1.18	–
Control_M	2.99	0.88	0.61	2.83	0.84	0.73
Filial piety_M	4.16	0.72	0.72	3.93	0.77	0.83
Warmth_F	3.67	1.05	0.84	3.47	0.93	0.86
Independence_F	3.87	0.92	0.80	3.78	0.84	0.86
Rejection_F^#^	2.60	1.53	–	2.67	1.30	–
Control_F	2.80	1.00	0.72	2.69	0.97	0.80
Filial piety_F	4.07	0.80	0.77	3.85	0.77	0.81
Warmth_P	3.91	0.72	0.70	3.85	0.67	0.72
Independence_P	4.09	0.62	0.73	4.06	0.61	0.82
Rejection_P	2.01	0.77	0.60	1.89	0.66	0.70
Control_P	3.30	0.83	0.70	3.23	0.73	0.73
Filial piety_P	4.20	0.55	0.71	4.17	0.57	0.81

*IR, Interpersonal Relatedness. The letter following the variable name refers to the resource of the corresponding data: M, self-reported data for maternal parenting style; F, self-reported data for paternal parenting style; P, parent-reported; ^#^, the subscale is composed of one item and the alpha value is not computed*.

### Procedure

The same procedure was used in both waves of data collection. The students were administered self-report measures of IR and parenting style. The students completed the questionnaires in their classrooms in ~30 min. Each participant received a small gift worth ~20 RMB for their participation. The students were told that the data are collected for scientific research and will be kept confidential and that data analysis will be performed at the group level.

As for the parental data collection, students took the corresponding parental questionnaires to their parents, and then one parent of each student completed the questionnaires at home, which were brought back to school in a sealed envelope and given to the research assistant. Written consent was obtained from the participants and their parents through the school. This study was approved by the ethics panel of the university.

### Data Analysis

The metric invariance is a prerequisite for comparing associations across time (Klimstra et al., 2018). In order to conduct cross-lagged analyses, a metric invariance of the measures of parenting styles (except for adolescent-reported rejection which included one item) and personality was examined. Items for the dimensions with eight items (i.e., parent-reported filial piety and IR) were parceled into three parcels using random assignment (Little et al., 2002). In general, the results indicated metric invariance in the measures of parenting styles and personality, which allowed us to conduct further cross-lagged analyses.

A repeated measures MANOVA was conducted to examine the effects of time, parental gender, and adolescent gender on the research variables. Cross-lagged analyses were conducted to examine reciprocal longitudinal associations between parenting styles and personality variables using Mplus (Version 7.4), which used full information maximum likelihood (FIML) estimation in the presence of missing data. Robust maximum likelihood estimation (MLR) was used to account for non-normal distributions of the observed variables.

In case of common method bias, Harman's single factor was used to test the potential limitation of the self-reported parenting styles and IR. The total variance for a single factor was 23.43%, which is acceptable (Podsakoff et al., 2003).

## Results

### Preliminary Analysis

The descriptive statistics of the research variables are reported in Table 1. The zero-order correlations between parenting styles and IR traits in Grades 6 and 8 are reported in Table 2. Parental education level and income level were included in the correlational analysis as indicators of family socioeconomic status.

**Table 2:** Zero-order correlations among the research variables.

	**1**	**2**	**3**	**4**	**5**	**6**	**7**	**8**	**9**	**10**	**11**	**12**	**13**	**14**	**15**	**16**	**17**	**18**	**19**	**20**
1. Edu_F	–	–	–	–	0.11*	0.07	0.12*	−0.10	−0.09	0.06	0.13*	0.21**	−0.10	−0.06	0.13*	0.19**	0.19**	−0.18**	−0.10	0.08
2. Edu_M	0.66**	–	–	–	0.05	0.06	0.09	−0.08	−0.08	0.00	0.07	0.09	−0.05	−0.11*	−0.04	0.12*	0.13*	−0.16**	−0.11*	0.02
3. Income_F	0.26**	0.22**	–	–	−0.01	0.05	0.13*	−0.12*	−0.02	0.03	0.05	0.12*	−0.03	0.02	0.04	0.11*	0.08	−0.06	−0.05	0.02
4.Income_M	0.22**	0.33**	0.42**	−−	0.02	−0.02	0.05	−0.15**	0.03	0.00	0.01	0.02	−0.03	−0.01	−0.03	0.12*	0.15**	−0.03	−0.05	0.08
5. IR	−0.05	−0.01	−0.02	0.02	–	0.32**	0.30**	−0.26**	−0.23**	0.15**	0.38**	0.27**	−0.31**	−0.22**	0.18**	0.24**	0.22**	−0.25**	0.02	0.20**
6. Warmth_M	0.05	0.06	0.01	0.04	0.38**	–	0.76**	−0.27**	−0.04	0.43**	0.58**	0.51**	−0.14**	−0.01	0.32**	0.27**	0.23**	−0.12*	−0.01	0.08
7. Independence_M	0.08*	0.11**	0.09*	0.07	0.33**	0.63**	–	−0.25**	−0.05	0.51**	0.50**	0.64**	−0.18**	−0.07	0.40**	0.19**	0.18**	−0.13**	−0.08	0.04
8. Rejection_M	−0.03	−0.01	0.00	−0.08*	−0.18**	−0.29**	−0.16**	–	0.38**	0.09	−0.17**	−0.13**	0.33**	0.28**	0.02	−0.12*	−0.12*	0.13**	0.00	−0.04
9. Control_M	0.01	−0.01	−0.01	−0.09*	−0.07	0.01	0.03	0.28**	–	0.32**	0.09	0.01	0.33**	0.55**	0.25**	−0.01	−0.04	0.09	0.12*	−0.01
10. Filial piety_M	0.01	−0.03	0.04	−0.07	0.25**	0.41**	0.41**	0.01	0.24**	–	0.31**	0.42**	0.04	0.11*	0.65**	0.09	0.04	−0.04	−0.04	0.05
11. Warmth_F	0.05	−0.01	0.06	0.02	0.29**	0.53**	0.43**	−0.13**	0.12**	0.36**	–	0.71**	−0.30**	0.07	0.41**	0.23**	0.15**	−0.13**	0.02	0.07
12. Independence_F	0.14**	0.09*	0.12**	0.05	0.27**	0.43**	0.59**	−0.10**	0.10**	0.43**	0.64**	–	−0.25**	−0.03	0.59**	0.17**	0.15**	−0.14**	−0.03	0.05
13. Rejection_F	−0.03	−0.01	−0.02	−0.03	−0.23**	−0.12**	−0.09*	0.36**	0.23**	−0.02	−0.25**	−0.21**	–	0.52**	0.08	0.01	0.01	0.08	0.01	−0.03
14. Control_F	−0.02	−0.07	−0.02	−0.01	−0.16**	0.07	0.06	0.19**	0.56**	0.10*	0.07*	0.04	0.38**	–	0.21**	−0.05	0.00	0.18**	0.12*	0.00
15. Filial piety_F	0.02	−0.04	0.02	−0.04	0.20**	0.33**	0.38**	0.00	0.18**	0.66**	0.40**	0.50**	0.03	0.25**	–	0.08	0.05	−0.06	0.02	0.07
16. Warmth_P	0.09*	0.14**	0.10*	0.08	0.19**	0.19**	0.09*	−0.07	−0.10*	0.06	0.14**	0.08*	−0.09*	−0.14**	−0.04	–	0.70**	−0.30**	0.22**	0.55**
17. Independence_P	0.08*	0.10**	0.08*	0.08*	0.14**	0.12**	0.13**	−0.10**	−0.06	0.06	0.06	0.09*	−0.02	−0.08*	0.05	0.59**	–	−0.26**	0.26**	0.61**
18. Rejection_P	−0.06	−0.09*	0.04	−0.01	−0.18**	−0.17**	−0.09*	0.13**	0.13**	−0.03	−0.13**	−0.11**	0.15**	0.14**	−0.03	−0.35**	−0.27**	–	0.19**	−0.08
19. Control_P	−0.07	−0.08*	0.00	−0.05	−0.01	−0.08*	−0.07	0.09*	0.08*	0.03	−0.03	−0.03	0.06	0.03	−0.01	0.09*	0.11**	0.22**	–	0.49**
20. Filial piety_P	−0.03	0.00	0.06	−0.03	0.07	0.01	−0.02	0.00	0.02	0.08*	0.03	0.03	−0.01	−0.03	0.03	0.42**	0.48**	−0.11**	0.38**	–

IR, Interpersonal Relatedness. The letter following the variable name refers to the resource of the corresponding data: M, adolescent-rated maternal parenting style; F, adolescent-rated paternal parenting style; P, parent-rated parenting style. Edu_F, father's education level; Edu_M, mother's education level; Income_F, father's income level; Income_M, mother's income level. Correlations of the variables at Grade 6 are presented below the diagonal line. Correlations of the variables at Grade 8 are presented above the diagonal line (Edu_F, Edu_M, Income_F, and Income_M were collected in Grade 6). ^*^p < 0.05; ^**^p < 0.01.

MANOVA indicated no significant differences in IR and self-rated parenting styles between the students for whom two waves of data collection were completed and those who did not participate in Grade 8, Wilks' Λ = 0.97, *F*_(11, 688)_ = 1.80, *p* > 0.05. MANOVA also indicated no significant differences in parent-rated parenting styles between those parents for whom two waves of data collection were completed and those who did not participate in Grade 8, Wilks' Λ =.99, *F*_(5, 404)_ = 0.75, *p* > 0.05.

For student-rated variables, the overall effects of time, parental gender, and adolescent gender as well as their interactions were examined with a mixed repeated measure MANOVA. According to Cohen's (1988) guidelines for interpreting the *F*-test effect size (small, 0.01; medium, 0.059; large, 0.138), only time differences, Wilks' Λ = 0.91, *F*_(5, 385)_ = 7.77, *p* = 0.00, η^2^ = 0.09, and parental gender differences, Wilks' Λ = 0.84, *F*_(5, 385)_ = 14.82, *p* = 0.00, η^2^ = 0.16, were considered in the subsequent analyses. Univariate tests indicated that maternal behavioral control was significantly higher than paternal behavioral control, with a medium effect size, *F*_(1, 389)_ = 31.00, *p* = 0.00, η^2^ = 0.07. A significant decrease was observed in parental encouragement of filial piety from Grade 6 to Grade 8, with a medium effect size, *F*_(1, 389)_ = 27.41, *p* = 0.00, η^2^ = 0.07. No other significant main effects or interaction effects were observed.

For parent-rated variables, the overall effects of time, parental gender, and adolescent gender as well as their interactions were examined with a mixed repeated measure MANOVA. According to Cohen's (1988) guidelines, only parental gender differences, Wilks' Λ = 0.92, *F*_(5, 172)_ = 3.08, *p* = 0.01, η^2^ = 0.08, and the time × adolescent gender interaction, Wilks' Λ = 0.93, *F*_(5, 172)_ = 2.59, *p* = 0.03, η^2^ = 0.07, were considered for further analyses. Univariate tests indicated non-significant differences.

### Cross-Lagged Analyses of Parenting Styles and IR

**Figure 1:**
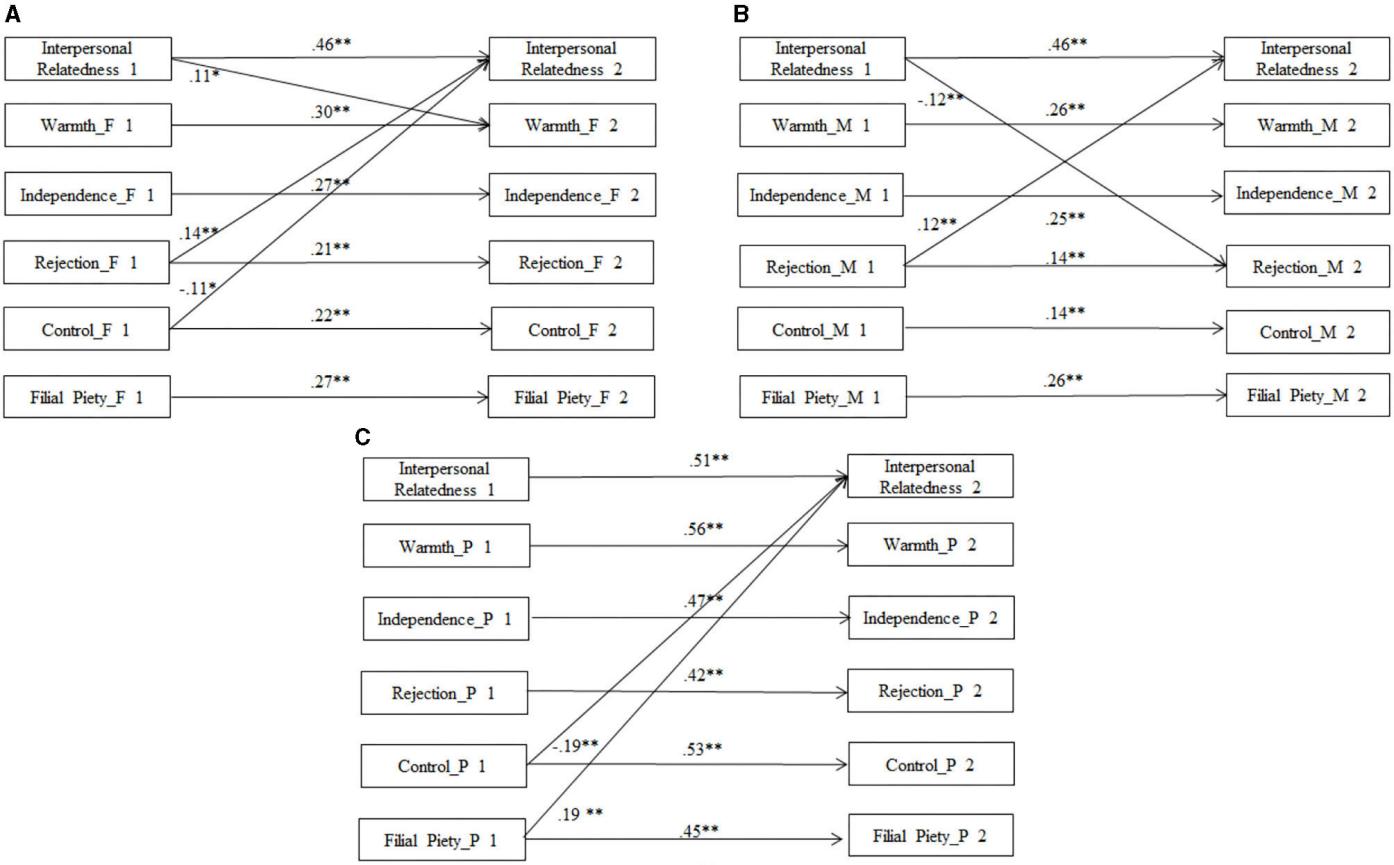
Cross-lagged model of IR and parenting styles from grade 6 to grade 8: **(A)** paternal styles rated by adolescents, **(B)** maternal styles rated by adolescents, and **(C)** parenting style rated by parents. For clarity, the non-significant paths in within-time correlations are not reported. The figure following the variable name refers to the time point of data collection in this study. F, father; M, mother; P, parent. *N* is 652 for Model **(A)**, 615 for Model **(B)**, and 396 for Model **(C)**. *p < 0.05; **p < 0.01.

Three cross-lagged models were tested to examine reciprocal longitudinal associations between parenting styles (including both student-rated and parent-rated) and IR (Figure 1). The cross-lagged relations between student-reported paternal and maternal parenting styles and IR were examined using Models 1 and 2, respectively, after autoregressive paths and the effects of adolescent gender and SES were controlled. Model 3 examined the relations between parent-reported parenting styles and IR. Data on parental styles reported by mothers and fathers were combined to increase the sample size that was needed for analyses, and parental gender was included as the third control variable in Model 3. Within-time correlations were also included in the three models.

In the final models, the non-significant paths from the control variables to IR and parenting styles were deleted. The data exhibited a suitable fit with Model 1 (χ^2^ = 84.45, *df* = 61, CFI = 0.98, RMSEA = 0.024, SRMR = 0.041), Model 2 (χ^2^ = 83.02, *df* = 64, CFI = 0.98, RMSEA = 0.022, SRMR = 0.038), and Model 3 (χ^2^ = 86.97, *df* = 57, CFI = 0.96, RMSEA = 0.036, SRMR = 0.064). The results of Model 1 indicated a significant positive effect of self-reported paternal rejection on IR in Grade 6 and a negative effect of paternal behavioral control on IR in Grade 8. IR in Grade 6 positively predicted paternal warmth in Grade 8. The results of Model 2 indicated a significant positive effect of self-reported maternal rejection in Grade 6 on IR in Grade 8. IR in Grade 6 negatively predicted maternal rejection in Grade 8. The results of Model 3 supported a significant negative effect of parent-reported behavioral control and a significant positive effect of parent-reported filial piety style in Grade 6 on IR in Grade 8.

We conducted multigroup analyses to test whether the cross-lagged paths between parenting styles and IR were significantly moderated by adolescent gender in Models 1 and 2 and by adolescent gender and parental gender in Model 3. Significant differences would be indicated between models when at least two of the following three criteria were met: Δχ^2^ significant at *p* < 0.05, ΔCFI ≥ −0.01, and ΔRMSEA ≥ 0.015 (Negru-Subtirica et al., 2015). The results for Model 1 [Δχ${}_{\left(10\right)}^{2}$ = 8.80, *p* > 0.05, ΔCFI = 0.00, ΔRMSEA = −0.002] and Model 2 [Δχ${}_{\left(10\right)}^{2}$ = 4.37, *p* > 0.05, ΔCFI = 0.01, ΔRMSEA = 0.001] indicated that the unconstrained model in which parameters were free to vary across groups was not significantly different from the constrained model in which parameters were fixed across adolescent genders. The results for Model 3 indicated that the unconstrained model in which parameters were free to vary across groups was not significantly different from the constrained model in which parameters were fixed across adolescent genders [Δχ${}_{\left(10\right)}^{2}$ = 0.95, *p* > 0.05, ΔCFI = 0.01, ΔRMSEA = −0.005] and parental genders [Δχ${}_{\left(10\right)}^{2}$ = 0.97, *p* > 0.05, ΔCFI = −0.00, ΔRMSEA = −0.001]. Therefore, both adolescent gender (for the three models) and parental gender (for Model 3) did not significantly moderate the cross-lagged relations.

## Discussion

This longitudinal study explored the associations between parenting styles and adolescents' IR personality, mainly from the perspectives of the internal working model (Bowlby, 1980; Bretherton, 1990) and the environmental elicitation model (Belsky, 1984; Shiner and Caspi, 2003). We examined the associations of IR personality with parenting styles based on adolescents' reports and parental reports. Moreover, we included dimensions of personality and parenting styles that are relevant in the Chinese society, which may help achieve a better understanding of the relations between parenting and personality in cultural contexts.

### Adolescent-Rated Parenting Styles and Adolescents' IR

The results showed that adolescents rated maternal behavioral control as higher than paternal behavioral control. The results seem to support the argument that mothers have traditionally been regarded as primary caregivers in Chinese families and the findings that mothers are involved in various aspects of socialization (Zhao et al., 2015; Lan et al., 2019). It is possible that relative to fathers, mothers spend more time with children and have more opportunities to monitor and control adolescent's behaviors (Chen et al., 2000). The results also indicated a decrease in parental encouragement of filial piety over the 2 years. One possible explanation is that although filial piety is highly valued in China (Cheung et al., 2001), the primary task of students in junior high school, especially in the final year, is to concentrate on academic performance to prepare for the entrance examinations for senior high school, which is linked to opportunities to receive a higher education (Zhao, 2007). As such, when students are in Grade 8, parents may be more concerned about their academic work and pay less attention to learning of filial behaviors.

The results indicated that paternal and maternal rejection reported by students positively predicted their IR. This finding is somewhat surprising because empirical studies often showed that parental rejection negatively predicted adolescents' empathy (Guo and Feng, 2017) and social relationships (Feldman and Downey, 1994). Apparently, further research is needed to explore the nature of parental rejection in the Chinese context. For example, it is possible that adolescents who perceive parental rejection may be more eager to establish social relationships in non-family contexts than adolescents do not. Consistent with this argument, Qiu (2010) found that children were likely to have earlier intimate relationships when perceiving higher parental rejection than those who did not perceive such parenting style. Rowe et al. (2015) also found positive effects of perceived parental rejection on adolescents' rejection sensitivity, which is a part of social sensitivity related to the trait of IR.

The child-reported paternal behavioral control negatively predicted IR, which also indicated the contribution of parenting to personality development. This result is consistent with previous finding that parental control positively predicted social fearfulness and difficulties in navigating social relationships in children (Rubin et al., 1999; Wood et al., 2006) and reduced adolescents' agreeableness (Van Heel et al., 2019), which is related to IR (Cheung et al., 2008; Fan et al., 2011). According to the self-system model of motivational development, a controlling parent-child relationship may disrupt children's self-system development and undermine their efforts toward relatedness (Grolnick, 2002).

Concerning contributions of personality to parenting, we found that adolescents' IR positively predicted paternal warmth and maternal rejection. The results were consistent with the findings of previous studies that adolescent benevolent (agreeable) characteristics are helpful to develop positive parent-child interactions through eliciting harmonious social interactions and avoidance of conflict (Cheung et al., 2008; Fan et al., 2011; Egberts et al., 2015). The bidirectional relations between IR and parenting may reflect adolescents' self-regulation in parent-child relationship as a form of active adaptation.

### Parent-Rated Parenting Styles and Adolescents' IR

Results based on parent-rated parenting styles supported the working model. Parent-rated behavioral control negatively predicted adolescents' IR. The negative contribution of child-rated parental behavioral control to IR was consistent with the results concerning child-reported paternal behavioral control. Interestingly, parental encouragement of filial piety, which represents a set of culturally relevant virtues in Chinese culture, positively predicted IR. As indicated earlier, filial piety requires children to care for parents and elders in the family and to maintain family harmony (Chen, 2014; Jorgensen et al., 2017). The children of parents who emphasize filial piety may be more likely to develop positive interpersonal relationships than children of parents who do not emphasize filial piety.

In summary, our study did not show consistent results concerning the relations of parenting styles reported by children and parents with adolescents' personality. Such inconsistency has been observed by other researchers. For example, Tuvblad et al. (2013) found that the influence of youth's psychopathic personality on parental negative affect toward the child was found based on parental reports but not youth reports. García et al. (2006) also found differences in the relations among personality, parenting styles, and socialization outcomes based on parents' and children's reports. It is possible the two sources of information represent different perspectives. Whereas adolescents' reports focus on their perceptions of parenting, which is relevant to their reactions (Shelton et al., 1996), parental reports may be more sensitive in capturing the unobservable and complex parental attitudes and behaviors in interactions with their children (Bezdjian et al., 2011). Our study showed that it may be useful to use both parent- and child-reports to obtain more complete information in the study of parenting and adolescents' characteristics.

### Conclusions, Implications, and Limitations

The present study provided valuable information about the associations between parenting styles and interpersonal personality in Chinese adolescents. A bi-directional relation between parenting and child characteristics has been highlighted in socialization theories (Bell, 1968; Belsky, 1984; Bronfenbrenner, 1989; Dodge, 1990). The results, specifically, indicated reciprocal contributions of parenting and adolescent IR personality, supporting both the working model (Bretherton, 1990) and the environmental elicitation model (Shiner and Caspi, 2003). Our results also suggest that paternal and maternal styles may have different effects on adolescents' IR personality. In addition, the data from multiple informants allowed us to examine parenting styles from adolescent and paternal perspectives and their relations with IR personality.

The results of the present study, especially those related to the culturally relevant aspects of parenting and personality, have important implications in theory and practice. For example, beyond the bidirectional associations between the major personality traits and parenting dimensions that are identified in the literature (e.g., Van den Akker et al., 2014), our study highlighted the relevance of indigenous constructs of parenting and personality, such as encouragement of filial piety and IR in the Chinese context. Practically, the results concerning the bi-directional relations between parenting and IR personality suggest that it is important to consider the dynamic process of parent-adolescent interactions when designing prevention and intervention programs for adolescents with adjustment problems.

Specifically, the elicited effects of adolescents' IR personality on parenting suggest that intervention programs designed to improve parenting should consider adolescents' characteristics. For example, the one-child policy, which started in the late 1970s, has resulted in concerns about indulgence of Chinese parents in childrearing (e.g., Liang and Sugawara, 1992; Chen et al., 2000). The formation of parental indulgence may be related to parental attitude as well as the characteristics of children. The unique personality traits of only children in China (Cameron et al., 2013; Love et al., 2020) may play a role in shaping parenting styles to a certain extent. It will be useful to help parents understand the characteristics of children's personality in order to effectively promote healthy development.

Several limitations and weaknesses in the study should be noted. First, we used a two-wave longitudinal design. A multi-wave longitudinal study should be conducted to explore the relations between parenting styles and adolescent personality over a longer period of time. Second, our sample included secondary school students in Shanghai, which is a fast-developing city in China. Parenting behaviors may be different from those in other regions, particularly rural regions, in the country where traditional values may be more maintained in childrearing. Thus, future research should be conducted with samples from different regions in China. Relatedly, it will be important to conduct cross-cultural research to examine whether the results of the present study concerning the relations between personality and parenting styles can be generalized to other cultures. Third, because of the limited parent-rated data at Time 2, data from maternal and paternal reports of parenting styles were combined when exploring their relations with IR after controlling for parental gender. It will be important in the future to examine how parenting styles as reported by mothers and fathers separately are associated with adolescent personality in a larger sample. Finally, given that adolescence is the period of rapid development of meta-cognitive abilities, which may affect the accuracy of self-reports (Shaughnessy et al., 2008), future research should assess meta-cognition when using self-report methods in adolescent studies.

## Data Availability Statement

The raw data supporting the conclusions of this article will be made available by the authors, without undue reservation.

## Ethics Statement

The studies involving human participants were reviewed and approved by Shanghai Normal University. Written informed consent to participate in this study was provided by the participants' legal guardian/next of kin.

## Author Contributions

WF, ML, and XC substantially contributed to the conception and the design of the work and interpreted the data and prepared the draft and finalized it. WF and ML contributed to the acquisition of data. ML analyzed the data. All authors approved the final version of the manuscript for submission.

## Funding

This research was supported by the Project of the Development of Career Education for Middle School Students in Shanghai (SHNU).

## Conflict of Interest

The authors declare that the research was conducted in the absence of any commercial or financial relationships that could be construed as a potential conflict of interest.

## Publisher's Note

All claims expressed in this article are solely those of the authors and do not necessarily represent those of their affiliated organizations, or those of the publisher, the editors and the reviewers. Any product that may be evaluated in this article, or claim that may be made by its manufacturer, is not guaranteed or endorsed by the publisher.

## Appendix

**Appendix 1:** Measurement invariance tests of parenting style and IR scales.

	**Model**	**χ^2^**	***df***	**RMSEA**	**CFI**	**Δχ^2^ (Δ*df*)**	**ΔRMSEA**	**ΔCFI**
Adolescent-reported	Configural invariance	169.791	34	0.065	0.956			
maternal warmth	Metric invariance	183.684	39	0.062	0.953	13.893^*^ (5)	0.003	0.003
Adolescent-reported	Configural invariance	115.656	53	0.035	0.972			
maternal independence	Metric invariance	119.436	59	0.033	0.973	3.78 (6)	0.002	0.006
Adolescent-reported	Configural invariance	80.400	34	0.038	0.954			
maternal control	Metric invariance	83.279	39	0.034	0.956	2.879 (5)	0.004	0.002
Adolescent-reported	Configural invariance	181.785	53	0.050	0.947			
maternal filial piety	Metric invariance	200.588	59	0.050	0.942	18.803^**^ (6)	0.000	0.005
Adolescent-reported	Configural invariance	186.226	34	0.069	0.951			
paternal warmth	Metric invariance	196.625	39	0.065	0.949	10.399 (5)	0.004	0.002
Adolescent-reported	Configural invariance	139.447	53	0.041	0.969			
paternal independence	Metric invariance	147.814	59	0.040	0.968	8.367 (6)	0.001	0.001
Adolescent-reported	Configural invariance	82.174	34	0.039	0.972			
paternal control	Metric invariance	82.998	39	0.034	0.974	0.824 (5)	0.005	0.002
Adolescent-reported	Configural invariance	119.347	53	0.036	0.977			
paternal filial piety	Metric invariance	123.558	59	0.034	0.978	4.211 (6)	0.002	0.001
Parent-reported	Configural invariance	64.963	19	0.077	0.904			
warmth	Metric invariance	67.292	23	0.069	0.907	2.329 (4)	0.008	0.003
Parent-reported	Configural invariance	160.921	53	0.071	0.868			
independence	Metric invariance	167.107	59	0.067	0.868	6.186 (6)	0.004	0.000
Parent-reported	Configural invariance	50.654	19	0.064	0.888			
rejection	Metric invariance	59.534	23	0.062	0.871	8.88 (4)	0.002	0.017^a^
Parent-reported	Configural invariance	71.412	19	0.082	0.885			
control	Metric invariance	76.062	23	0.075	0.884	4.65 (4)	0.007	0.001
Parent-reported	Configural invariance	29.524	8	0.081	0.945			
filial piety	Metric invariance	31.531	11	0.068	0.948	2.007 (3)	0.013	0.003
IR	Configural invariance	53.517	8	0.077	0.980			
	Metric invariance	55.483	11	0.065	0.980	1.966 (3)	0.012	0.000

*IR, Interpersonal Relatedness; CFI, Comparative Fit Index; RMSEA, Root Mean Square Error of Approximation.^*^p < 0.05;^**^p < 0.01.^a^ ΔCFI or ΔRMSEA higher than cutoffs*.
